# Determinants and risk prediction models for frailty among community-living older adults in eastern China

**DOI:** 10.3389/fpubh.2025.1518472

**Published:** 2025-03-11

**Authors:** Lin Qi, Jianyu Liu, Xuhui Song, Xinle Wang, Mengmeng Yang, Xinyi Cao, Yan He

**Affiliations:** ^1^College of Management, Hainan Medical University, Haikou, China; ^2^College of Public Health, Zhengzhou University, Zhengzhou, China

**Keywords:** frailty, community-dwelling older adults, random forest, XGBoost, Tilburg frailty indicator, risk prediction

## Abstract

**Objective:**

The purpose of this study is to develop predictive models for frailty risk among community-dwelling older adults in eastern China using machine learning techniques. This approach aims to facilitate early detection of high-risk individuals and inform the design of tailored interventions, with the ultimate goals of enhancing quality of life and mitigating frailty progression in the older adult population.

**Methods:**

This study involved 1,263 participants aged 60 years or older, who were selected through stratified cluster sampling. Frailty was assessed using the Tilburg Frailty Indicator (TFI), which encompasses physical, psychological, and social dimensions. Predictive models were constructed using decision trees, random forests, and XGBoost algorithms, implemented in R software (version 4.4.2). The performance of these models was evaluated using metrics such as the area under the receiver operating characteristic curve (AUC), ROC curves, and confusion matrices.

**Results:**

The results showed that 64.77% of the older adult were physically weak. Body mass index (BMI), living arrangements, frequency of visits and smoking status are the main factors contributing to frailty. When comparing predictive model metrics, random forest and extreme Gradient Lift (XGBoost) outperform decision tree models in terms of accuracy and applicability.

**Conclusion:**

Older adults living in communities in eastern China showed slight frailty, and many factors influenced their frailty scores. Random forest and XGBoost models outperform decision tree models in predicting frailty in older adults, so identifying high-risk individuals early and developing personalized interventions can help slow the development of frailty and improve quality of life in older adults.

## 1 Introduction

Frailty is marked by the decline of multiple physiological systems, reducing the ability to cope with external stressors and increasing the risk of adverse health outcomes (1–3). It is a significant challenge in aging populations and has become a major public health concern as global aging accelerates (4). Our study shows that frailty rates rise sharply with age, exceeding 20% among those aged 80 and older. Frailty affects individuals on physical, psychological, and social levels and is closely tied to chronic diseases like heart disease, diabetes, and respiratory conditions, which worsen frailty (5–8). Sarcopenia is also a key contributor (9), while mental health issues such as depression and anxiety, often linked to social isolation, further exacerbate frailty (10, 11). These multifaceted impacts increase health risks, reduce quality of life, and drive up healthcare costs, placing a heavy burden on families and society (12). Identifying frailty risk factors and developing effective predictive models are now central to geriatric research.

To better assess and prevent frailty, various tools have been developed. The Tilburg Frailty Indicator (TFI) is widely used, covering physical, psychological, and social dimensions through 15 items (13). Other tools, such as the Fried Frailty Phenotype and Frailty Index, are also used to identify high-risk individuals and guide interventions (14–16). In this study, we chose the TFI to assess frailty among older adult individuals in eastern China, capturing its multidimensional nature. Research shows that machine learning models like decision trees, random forests, and XGBoost are effective for predicting frailty risk. However, many studies lack systematic internal validation after model construction, limiting the evaluation of model stability and reliability. Even when validation is conducted, some studies use simpler methods, such as Bootstrap resampling without cross-validation (17–20). This study aims to conduct systematic internal validation, compare model performance, and select an optimal model with strong generalization and robustness for frailty risk prediction. Early detection and appropriate interventions, such as exercise, nutritional support, and psychological care, can significantly slow frailty progression and improve quality of life in older adults (21). Interventions should focus on managing reversible diseases and preventing frailty through early identification. This study uses a machine learning model to predict frailty risk, aiming to provide personalized intervention recommendations for middle-aged and older adult individuals in the community and offer a scientific basis for future public health policies.

## 2 Methods

### 2.1 Data collection

**Figure 1:**
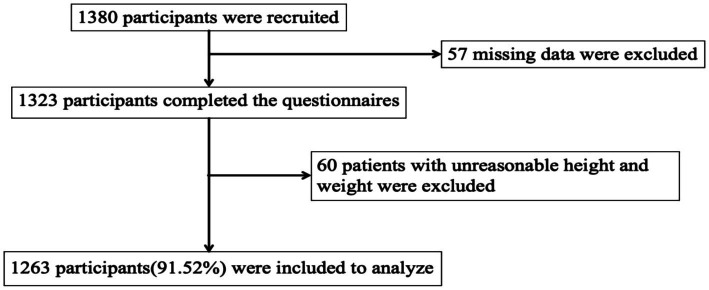
Flowchart on participant recruitment.

This study targeted older adults living in communities in eastern China and collected data through a cross-sectional survey conducted by public health staff between September and December 2021 as part of a household survey. The method of multi-stage stratified cluster sampling is adopted. The first step is to randomly select Shandong, Jiangsu and Guangdong from 12 provinces in eastern China. The second step is to randomly select one municipality from each selected province: Qingdao, Suzhou, and Guangzhou. Step 3: Jimo District of Qingdao, Kunshan County of Suzhou and Yuexiu District of Guangzhou shall set up one district or county each. The fourth step is to randomly select two streets from each district, and then select two neighborhoods from each street. Participants were randomly sampled from these communities. The inclusion criteria were older adult individuals aged 60 or older, with at least two chronic diseases diagnosed by a doctor, and those who consented to participate after being informed about the survey. Exclusion criteria included incomplete information on chronic disease conditions or responses with logical errors or unclear answers. Out of 1,380 collected questionnaires, 1,323 were retained after excluding those with missing key variables. Further exclusions were made for errors or unreasonable data related to height and weight, leaving 1,263 older adult individuals for the final analysis. The recruitment process of participants is shown in Figure 1.

### 2.2 Covariates and frailty assessment

The following factors are considered covariates. Demographic factors include gender, age, whether you live in rural or urban areas, race, education level, marital status, frequency of medical visits, living arrangements, monthly income, BMI, and pension insurance. Lifestyle and chronic disease factors included smoking status, drinking habits, number of chronic diseases, type of medication, length of sleep, and the frequency of participants’ outdoor activities. The study defined drinking status as “yes” for those who had consumed alcohol and were still drinking. People who have never drunk alcohol, and people who have stopped drinking are rated as “no.” Smoking is defined as “yes” for those who have smoked and are still smoking, “no” for those who have never smoked, and “no” for those who have quit smoking.

The Tilburg Frailty Indicator (TFI) was chosen to assess frailty in older adults. Developed by Gobbens et al. (13), this instrument integrates multiple frailty models to evaluate frailty status in the older adult (22). It comprises 15 items divided into three domains: physical, psychological, and social. The physical domain (8 items) covers conditions like poor health, unexplained weight loss, walking difficulties, balance issues, vision and hearing impairments, reduced grip strength, and persistent fatigue. The psychological domain (4 items) examines memory decline, depression, anxiety, and problem-solving difficulties. The social domain (3 items) assesses social isolation, lack of interpersonal relationships, and insufficient social support. The TFI uses a binary scoring system (1 point for frailty, 0 for no frailty; some items score 0.5). Scores range from 0 to 15, with ≥5 indicating frailty and higher scores reflecting greater severity. Participants were categorized as frail or non-frail based on these scores. The scale exhibited good internal consistency, with a Cronbach’s *α* of 0.72.

### 2.3 Principle and evaluation method of decision tree, random forest and XGBoost model

#### 2.3.1 Decision tree model

The decision tree is a tree-based model used for classification and regression. It recursively divides the dataset into smaller subsets to build the model. The fundamental concept involves selecting features that maximize data purity and using these features to split the data.

Formula:

Information entropy: used to measure the uncertainty of data, the formula is calculated as:


$$\mathrm{Entropy}\left(S\right)=-\overset{n}{\underset{i=1}{\Sigma }}p_{i}\log_{2}\left(p_{i}\right)$$

Where *pi* is the proportion of Class *i* samples in the data set.

Information gain: used to measure the effect of features on reducing uncertainty, calculated by:


$$\mathrm{Gain}\left(S,F\right)=\mathrm{Entropy}\left(S\right)-\underset{v\in Values\left(F\right)}{\Sigma }\frac{\left|S_{v}\right|}{\left|S\right|}\mathrm{Entropy}\left(S_{v}\right)$$

Where *F* is the feature, *Values(F)* is all possible values of feature *F*, and *Sv* is the subdataset of feature *F* with value *v*.

#### 2.3.2 Random forest model

Random forest enhances model accuracy and robustness by constructing multiple decision trees and aggregating their predictions via voting or averaging. It employs Bootstrap sampling to create subsamples from the dataset. Each subsample generates a decision tree, with random feature subsets selected at each split. The final prediction is obtained by consolidating the outputs of all trees. The construction of individual trees in random forest follows the principles of a standalone decision tree, using information gain or the Gini index to determine optimal splits.

#### 2.3.3 XGBoost model

XGBoost is a gradient-boosting-based ensemble algorithm that enhances model performance by iteratively adding decision trees. Each new tree is built to address the prediction errors of its predecessor, thereby progressively reducing model bias.

Formula:

The objective function of XGBoost is:


$$\mathrm{obj}\left(\theta \right)=\frac{1}{N}\overset{N}{\underset{i=1}{\Sigma }}L\left(y_{i},f\left(x_{i}\right)\right)+\Omega \left(f\right)$$

*L(yi, f(xi))* represents the loss function, which quantifies the discrepancy between the predicted and actual values. Meanwhile, Ω*(f)* serves as the regularization term to manage the model’s complexity.

When splitting a node, XGBoost determines whether to split by calculating the gain after splitting:


$$\mathrm{Gain}=\frac{1}{2}\left(\frac{G_{L}^{2}}{H_{L}+\lambda }-\frac{G_{R}^{2}}{H_{R}+\lambda }-\frac{{\left(G_{L}+G_{R}\right)}^{2}}{H_{L}+H_{R}+\lambda }-\gamma \right)$$

*GL* and *GR* denote the first-order derivatives of the left and right subtrees before and after splitting, respectively. *HL* and *HR* represent the sums of the second-order derivatives. Meanwhile, *λ* and *γ* are regularization parameters.

#### 2.3.4 Performance evaluation of prediction model

To evaluate the model’s performance comprehensively, the dataset was randomly split into training and test sets at a 7:3 ratio. A confusion matrix was generated based on this division to assess the model’s predictive accuracy. The key performance metrics calculated include Accuracy (ACC), Recall (R), Precision (P), F1 score, and AUC (Area Under the ROC Curve). The confusion matrix is detailed in Table 1.

**Table 1:** Confusion matrix of binary classification model.

True category	Prediction category	
	Negative	Positive
Negative	True Negative, TN	False Positive, FP
Positive	False Negative, FN	True Positive, TP

The calculation formula of each index is as follows:

(1)  Accuracy rate represents the probability that the prediction sample is correctly identified:

(1) $$\mathrm{ACC}=\frac{\mathrm{TP}+\mathrm{TN}}{\mathrm{TP}+\mathrm{FP}+\mathrm{TN}+\mathrm{FN}}$$

(2)  The recall rate represents the proportion of demand that is correctly predicted as a percentage of all real demand:

(2) $$R=\frac{\mathrm{TP}}{\mathrm{TP}+\mathrm{FN}}$$

(3)  The accuracy rate represents the true percentage of those who are really in demand:

(3) $$P=\frac{\mathrm{TN}}{\mathrm{FP}+\mathrm{TN}}$$

(4)  F1 score represents the harmonic mean of accuracy rate and recall rate, which can balance the relationship between recall rate and accuracy rate:

(4) $$F1=2\times \frac{R\times P}{R+P}$$

(5)  AUC represents the area under ROC curve. The AUC ranges from 0 to 1, with an AUC of 0.5 indicating no predictive power, 0.5 to 0.7 low predictive power, 0.7 to 0.9 medium predictive power, and higher than 0.9 high predictive power.

Given that accuracy, recall, and precision are single metrics and cannot fully capture model performance, this study uses both the F1 score and AUC value to provide a comprehensive assessment of the model’s predictive ability.

### 2.4 Statistical analysis

Statistical analyses were performed using SPSS 27.0, with significance set at *p* < 0.05. Categorical data were described using counts (*n*) and percentages (%), and chi-square tests were used to assess group differences. Variables significant in univariate analyses (*p* < 0.05) were included in binary logistic regression models to identify frailty-related factors. For predictive modeling, R 4.4.2 was employed with three machine learning packages: rpart for decision trees, randomForest for random forests, and xgboost for XGBoost. The dataset (*n* = 1,263) was split into training (*n* = 885, 70%) and testing sets (*n* = 378, 30%) to prevent overfitting and ensure model generalizability.

## 3 Results

### 3.1 Frailty status and demographic characteristics of community-dwelling older adults

**Figure 2:**
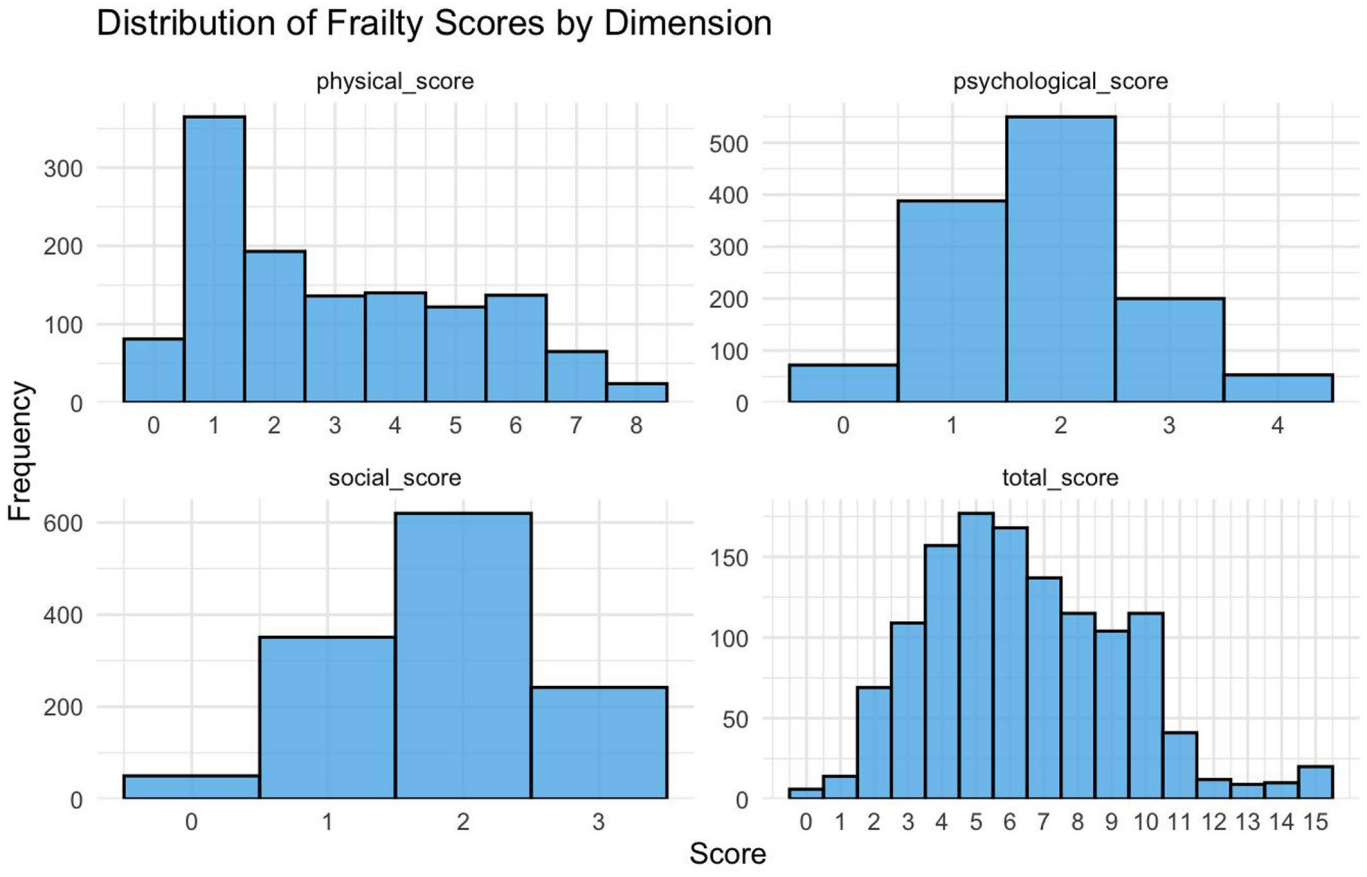
Overall frailty and various dimensions of frailty in the older adult.

According to the scores of the three frailty dimensions, most older adult individuals had physical scores between 0 and 3. This indicates that only a small number of them showed significant physical frailty. The psychological scores were mostly concentrated at 2, with fewer individuals scoring 0 (no psychological frailty) or 3–4. This suggests that most older adult individuals experienced mild to moderate psychological frailty. Similarly, social dimension scores were also centered at 2 points, with fewer older adult scoring 0 or 3. This implies that extreme social isolation or strong social support were uncommon. Overall, the majority of older adult individuals scored between 5 and 6, indicating that a higher percentage of community-dwelling older adult were mildly frail. Physical and social frailty were more noticeable, while psychological frailty, though less frequent, still needs attention (Figure 2).

### 3.2 Univariate and multivariate analysis of frailty risk factors

The study involved 1,263 older adult participants, comprising 47.5% males and 52.5% females. Their ages were distributed as follows: 24.2% were aged 60–69, 29.9% were 70–79, and 45.9% were 80 or older. The majority (97.1%) were Han Chinese, with 2.9% belonging to ethnic minorities. Educational backgrounds varied, with 18.0% illiterate, 34.7% having primary education, 21.3% with lower secondary education, and 26.0% holding high school or higher qualifications. Marital status showed that 50.4% were married, 11.4% were single, and 35.5% were widowed. Residence-wise, 55.7% lived in urban areas and 44.3% in rural areas. Regarding monthly income, 59.2% earned ≤3,000 yuan, while 60.7% had pension insurance. BMI data revealed that 9.9% were underweight, 65.7% were normal weight, 22.2% were overweight, and 2.2% were obese. Participants were categorized into frail (818) and non-frail (445) groups. Significant differences between the groups were observed in age, marital status, monthly income, pension insurance, BMI, living arrangements, visit frequency, smoking status, sleep duration, outdoor activity frequency, medication type, and number of chronic diseases (*p* < 0.05). The results indicate that older adults are more likely to be frail if they are older, widowed, have lower incomes, lack pension insurance, are underweight or overweight, live alone, have fewer visits, smoke, take multiple medications, or have multiple chronic conditions (Table 2).

**Table 2:** Demographic characteristics and single factor analysis of older adult people living at home in community.

Variables	Total population, *n* (%) (*n* = 1,263)	Frail, *n* (%) (*n* = 818)	Non-frail, *n* (%) (*n* = 445)	Chi-Square value (χ^2^)	*p* value
Gender				3.833	0.05
Male	600 (47.5)	372 (45.5)	228 (51.2)		
Female	663 (52.5)	446 (54.5)	217 (48.8)		
Age				31.972	<0.001
60–69	306 (24.2)	172 (21.0)	134 (30.1)		
70–79	377 (29.9)	223 (27.3)	154 (34.6)		
≥80	580 (45.9)	423 (51.7)	157 (35.3)		
Ethnicity				2.749	0.097
Han	1,227 (97.1)	790 (96.6)	437 (98.2)		
Ethnic minority	36 (2.9)	28 (3.4)	8 (1.8)		
Educational level				2.214	0.529
Illiterate	227 (18.0)	140 (17.1)	87 (19.6)		
Primary school	438 (34.7)	282 (34.5)	156 (35.1)		
Middle school	270 (21.3)	174 (21.3)	96 (21.6)		
High school and above	328 (26.0)	222 (27.1)	106 (23.8)		
Marital status				33.846	<0.001
Single	144 (11.4)	72 (8.8)	72 (16.2)		
Married	637 (50.4)	392 (47.9)	245 (55.1)		
Divorced	34 (2.7)	28 (3.4)	6 (1.3)		
Widowed	448 (35.5)	326 (39.9)	122 (27.4)		
Urban–rural status				1.062	0.303
Urban	703 (55.7)	464 (56.7)	239 (53.7)		
Rural	560 (44.3)	354 (43.3)	206 (46.3)		
Monthly income				5.437	0.02
≤3,000 RMB	748 (59.2)	465 (56.8)	283 (63.6)		
>3,000 RMB	515 (40.8)	353 (43.2)	162 (36.4)		
Pension Insurance				4.588	0.032
Yes	767 (60.7)	479 (58.6)	288 (64.7)		
No	496 (39.3)	339 (41.4)	157 (35.3)		
BMI				29.657	<0.001
Underweight	125 (9.9)	90 (11.0)	35 (7.9)		
Normal	830 (65.7)	561 (68.6)	269 (60.4)		
Overweight	280 (22.2)	159 (19.4)	121 (27.2)		
Obese	28 (2.2)	8 (1.0)	20 (4.5)		
Living arrangement				17.490	<0.001
Living alone	289 (22.9)	217 (26.5)	72 (16.2)		
Not living alone	974 (77.1)	601 (73.5)	373 (83.8)		
Visit frequency				21.890	<0.001
Frequent	658 (52.1)	443 (54.2)	215 (48.3)		
Sometimes	369 (29.2)	253 (30.9)	116 (26.1)		
Occasionally	236 (18.7)	122 (14.9)	114 (25.6)		
Smoking status				5.626	0.018
Yes	361 (28.6)	252 (30.8)	109 (24.5)		
No	902 (71.4)	566 (69.2)	336 (75.5)		
Alcohol consumption				2.369	0.124
Yes	248 (19.6)	171 (20.9)	77 (17.3)		
No	1,015 (80.4)	647 (79.1)	368 (82.7)		
Sleep duration				14.218	0.003
<4 h	43 (3.4)	30 (3.7)	13 (2.9)		
4–6 h	383 (30.3)	261 (31.9)	122 (27.4)		
6–8 h	655 (51.9)	431 (52.7)	224 (50.3)		
>8 h	182 (14.4)	96 (11.7)	86 (19.4)		
Outgoing frequency				27.338	<0.001
Frequent	783 (62.0)	466 (57.0)	317 (71.3)		
Sometimes	218 (17.3)	168 (20.5)	50 (11.2)		
Occasionally	262 (20.7)	184 (22.5)	78 (17.5)		
Types of medication				89.345	<0.001
0	159 (12.6)	65 (7.9)	94 (21.1)		
1	254 (20.1)	129 (15.8)	125 (28.1)		
≥2	850 (67.3)	624 (76.3)	226 (50.8)		
Number of chronic diseases				84.338	<0.001
0	122 (9.7)	46 (5.6)	76 (17.1)		
1	356 (28.2)	193 (23.6)	163 (36.6)		
≥2	785 (62.1)	579 (70.8)	206 (46.3)		

To identify factors associated with frailty, a binary logistic regression analysis was conducted. Variables significant in univariate analyses were included, with the last category serving as the reference group. Thirteen variables were examined, and collinearity diagnostics (VIF < 5) confirmed no multicollinearity. The analysis revealed that age, BMI, monthly income, living arrangement, visit frequency, pension insurance, smoking status, number of chronic diseases, and type of medication significantly predicted frailty (*p* < 0.05). Conversely, gender and marital status had no significant impact on frailty. Notably, BMI, living arrangements, visit frequency, and smoking status emerged as key risk factors. Overweight older adults were 5.139 times more likely to be frailty than those of normal weight (OR = 5.139, 95%CI = 2.111–12.507). Similarly, those who lived alone were 2.831 times more likely to be frail than those who did not (OR = 2.831, 95%CI = 1.958–4.092). Older adults who went out less frequently were 2.229 times more likely to be frail than those who went out more frequently (OR = 2.229, 95%CI = 1.509–3.294). Smokers are 1.443 times more likely to be weak than non-smokers (OR = 1.443, 95%CI = 1.034–2.015) (Table 3).

**Table 3:** Multifactor analysis of frailty of older adult people living at home in community.

Variables	*β*	Wald*χ*^2^	*p*	OR (95% CI)
Gender	−0.265	2.782	0.095	0.767 (0.562–1.047)
Age				
60–69	−0.464	6.013	0.014	0.628 (0.434–0.911)
70–79	−0.484	8.53	0.003	0.616 (0.445–0.853)
≥80		9.837	0.007	
BMI				
Underweight	1.57	10.094	0.001	4.807 (1.825–12.664)
Normal	1.637	13.008	<0.001	5.139 (2.111–12.507)
Overweight	1.23	7.004	0.008	3.422 (1.376–8.511)
Obese		17.855	<0.001	
Marital status				
Single	−0.029	0.011	0.916	0.971 (0.566–1.668)
Married	0.138	0.61	0.435	1.148 (0.812–1.625)
Divorced	0.879	2.944	0.086	2.407 (0.882–6.568)
Widowed		3.525	0.318	
Monthly income	−0.329	4.372	0.037	0.72 (0.529–0.98)
Living arrangement	1.041	30.64	<0.001	2.831 (1.958–4.092)
Visit frequency				
Frequent	0.802	16.195	<0.001	2.229 (1.509–3.294)
Sometimes	0.658	9.8	0.002	1.931 (1.279–2.916)
Occasionally		16.386	<0.001	
Pension insurance	−0.658	15.37	<0.001	0.518 (0.373–0.719)
Smoking status	0.367	4.648	0.031	1.443 (1.034–2.015)
Number of chronic diseases		23.325	<0.001	
0	−1.06	15.401	<0.001	0.346 (0.204–0.588)
1	−0.625	15.738	<0.001	0.535 (0.393–0.729)
≥2		23.325	<0.001	
Types of medication				
0	−0.616	6.717	0.01	0.54 (0.339–0.861)
1	−0.662	15.106	<0.001	0.516 (0.37–0.72)
≥2		17.459	<0.001	
Sleep duration				
<4 h	0.709	3.051	0.081	2.031 (0.917–4.499)
4–6 h	0.72	11.682	0.001	2.054 (1.359–3.104)
6–8 h	0.585	9.087	0.003	1.795 (1.227–2.627)
>8 h		12.771	0.005	
Outgoing frequency				
Frequent	−0.374	4.474	0.034	0.688 (0.487–0.973)
Sometimes	0.367	2.431	0.119	1.443 (0.91–2.288)
Occasionally		15.886	<0.001	

### 3.3 Application of decision tree, random forest, and XGBoost models in predicting frailty status of community-dwelling older adults

To enhance the performance of the prediction models and ensure comprehensive results, this study selected potential risk factors from univariate frailty analyses with *p* < 0.05 as the inclusion criterion. Thirteen independent variables were included for model construction. Three machine learning algorithms—decision tree, random forest, and XGBoost—were employed to predict frailty status among older adult individuals in eastern China. The dataset was split into 70% for training and 30% for testing.

**Figure 3:**
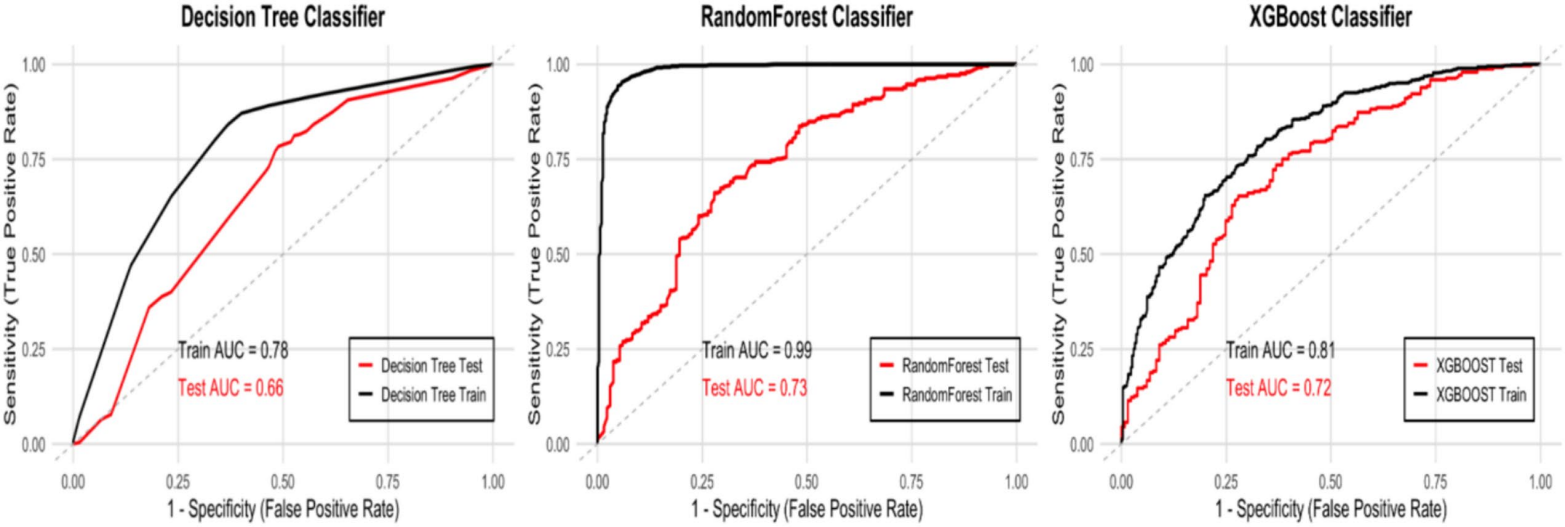
ROC curves and AUC values of the three models.

**Figure 4:**
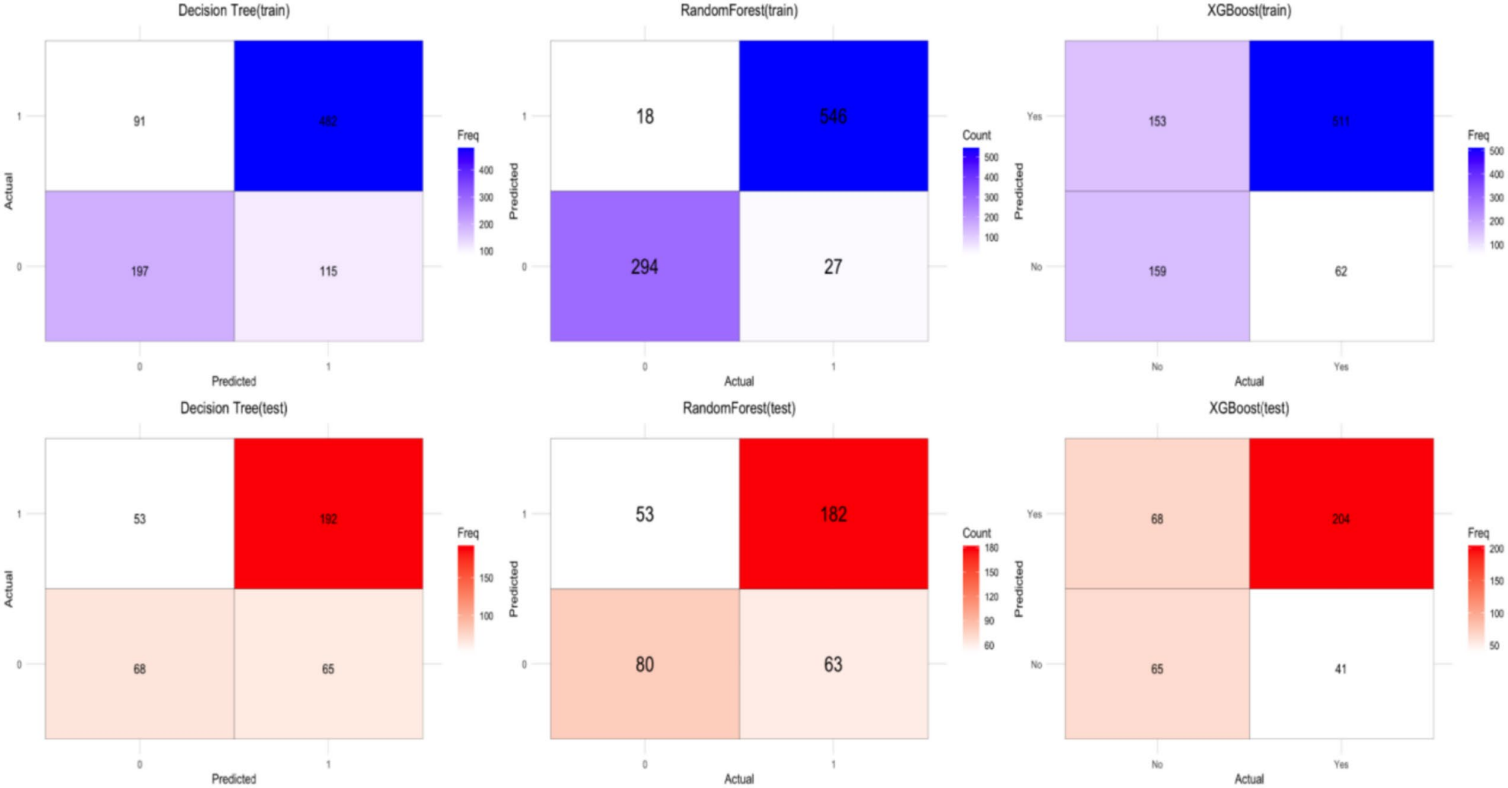
Confusion matrix results for the training set (top) and test set (bottom) of the three models.

The Decision Tree model uses recursive splitting to identify optimal features based on information gain or the Gini index. While interpretable, it is prone to overfitting, leading to poor generalization. In this study, the rpart package in R was used to build the model. To improve generalization, key parameters were optimized using grid search with the caret package. The optimal parameters were: (criterion = “gini”, max_depth = 7, min_samples_leaf = 3, min_samples_split = 8, splitter = “best”). The model achieved an AUC of 0.78 and an F1 score of 0.8239 on the training set, but the AUC dropped to 0.66 and the F1 score to 0.7649 on the test set, indicating some overfitting (Figures 3, 4).

Random forest improves robustness and accuracy by constructing multiple decision trees and aggregating predictions through majority voting, reducing the risk of overfitting. The randomForest package in R was used, with 5-fold cross-validation (method = “cv,” number = 5) and grid search for parameter tuning. The optimal parameters were: (ntree = 500, mtry = 2, nodesize = 5). The model achieved an AUC of 0.99 and an F1 score of 0.9604 on the training set, but the AUC dropped to 0.73 and the F1 score to 0.7583 on the test set, revealing significant overfitting (Figures 3, 4).

XGBoost enhances predictive performance by training and combining weak classifiers. It is efficient for large datasets, handles missing values, and mitigates overfitting through regularization. The xgboost package in R was used, with 5-fold cross-validation and grid search for parameter tuning. The optimal parameters were: (nrounds = 100, max_depth = 4, eta = 0.1). The model achieved an AUC of 0.81 on the training set and 0.72 on the test set, with an F1 score of 0.7986 on the training set and 0.7595 on the test set, indicating a slight overfitting issue (Figures 3, 4).

### 3.4 Comparison of model performance and feature importance analysis

Machine learning algorithms, whether single or ensemble-based, are widely utilized in the medical field. In studies predicting frailty risk among older populations, data are often collected through questionnaires. Given the relatively small sample size and numerous variables, this study employs both common single algorithms and ensemble algorithms to construct a binary classification prediction model. These include decision tree, random forest, and XGBoost. The performance of these models was evaluated on a test set, as detailed in Table 4.

**Table 4:** Comparison of three model evaluation indexes.

Models	Evaluation metrics					
	Accuracy	Recall	Precision	F1 score	AUC	Youden’s index
Decision tree	0.6878	0.7837	0.7471	0.7649	0.6593	0.2950
Random forest	0.6931	0.7429	0.7745	0.7583	0.7350	0.3444
XGBoost	0.6984	0.7347	0.6132	0.7595	0.7204	0.3749

According to Table 4, the random forest model achieved an AUC of 0.7350 and an F1 score of 0.7583, outperforming the XGBoost model, which had an AUC of 0.7204 and an F1 score of 0.7595. The Decision Tree model had the lowest AUC at 0.6593, with an F1 score of 0.7649. While random forest exhibited the best performance, it also showed significant overfitting, likely due to its complexity in capturing noise in the training data. Overall, both random forest and XGBoost models performed better than the Decision Tree model, mainly due to their higher AUC values.

**Figure 5:**
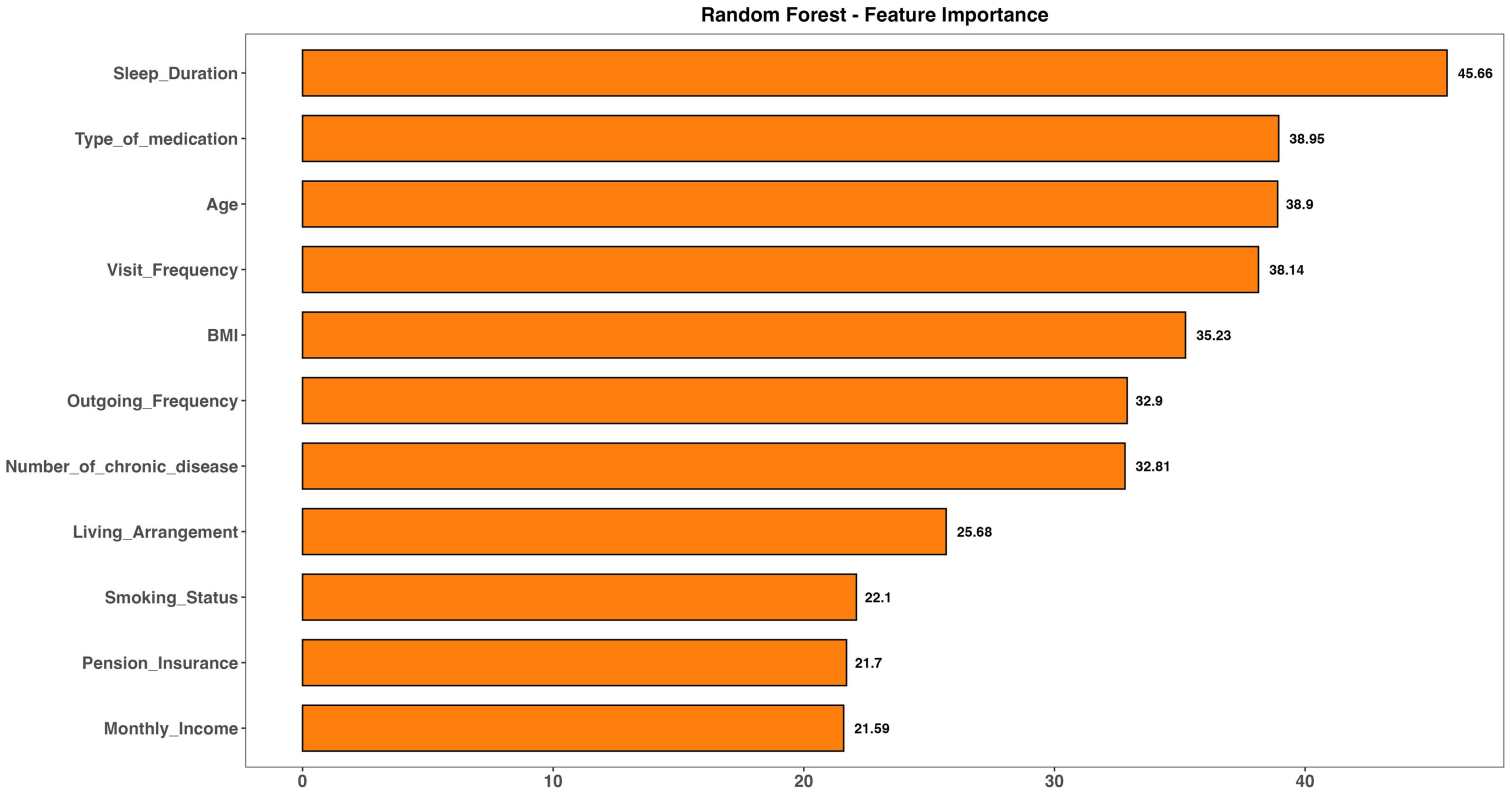
Visualize the feature importance in random forest model.

**Figure 6:**
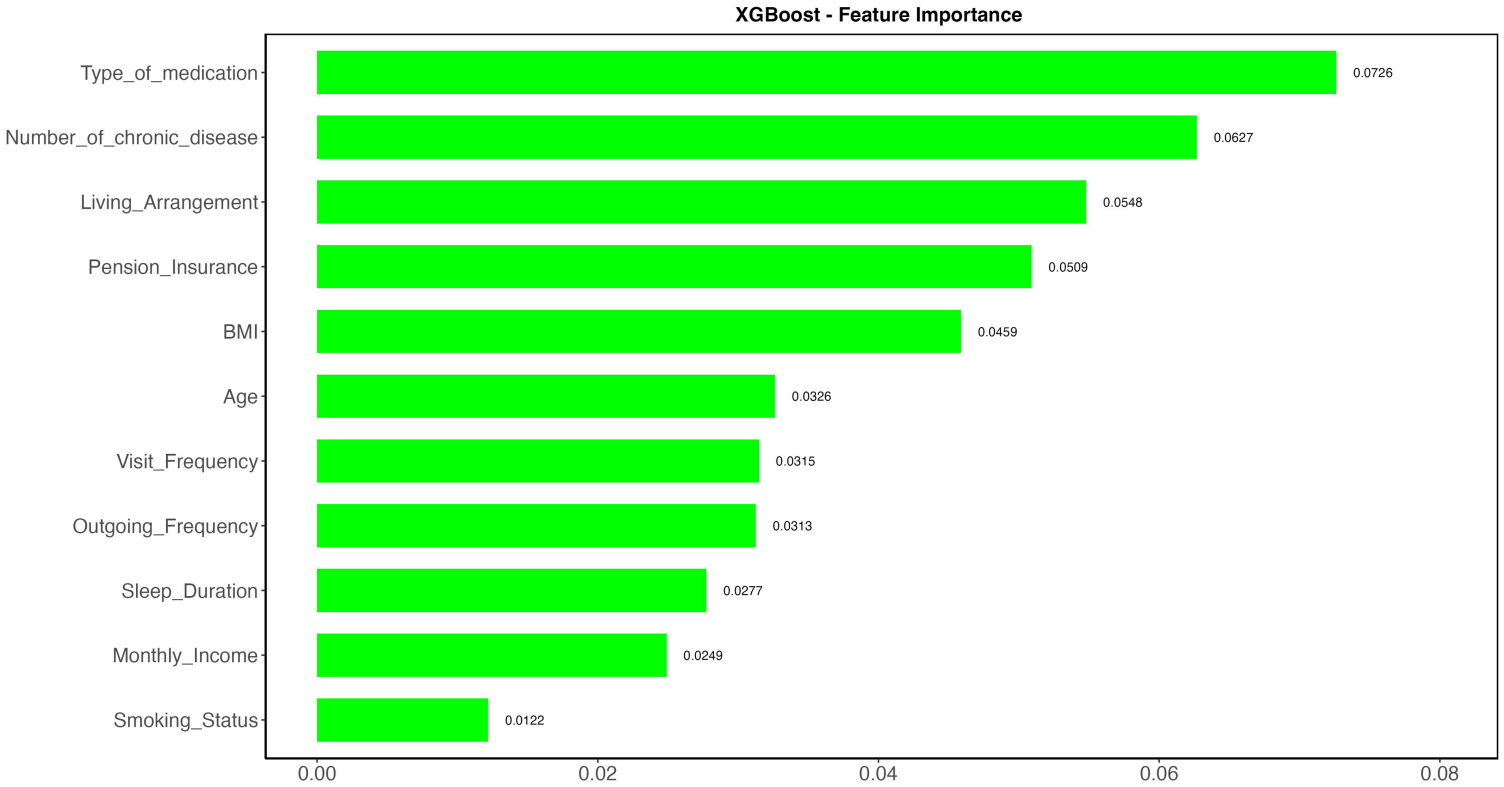
Visualize the feature importance in XGBoost model.

To further analyze model performance, the importance and xgb.importance functions in R were used to rank and visualize variable importance in both the random forest and XGBoost models. As depicted in Figures 5, 6, “Type of Medication” emerged as a key predictor of frailty risk in both models. Although the feature importance scores varied slightly, “Living Arrangement,” “Number of Chronic Diseases,” and “Sleep Duration” were also identified as significant factors. Given that XGBoost and random forest models excel at capturing complex and non-linear relationships, these factors should be prioritized in managing the daily lives of the older adult.

## 4 Discussion

In this study, among the 1,263 older adult participants, 818 were diagnosed with frailty syndrome, while 445 were not, yielding a frailty prevalence of 64.77% among community-dwelling older adult in eastern China. When compared to international studies, the pre-frailty rate was 50.9% in Northern Thailand (23), 55.5% in Indonesia (24), and 21% of participants in Australia were frail, with an additional 48% in the pre-frailty stage (25). The prevalence rates in this study are higher than those reported in other regions. This difference may be due to the use of the Tilburg Frailty Indicator, which assesses physical, psychological, and social dimensions, while other studies often rely on the Fried frailty phenotype, focusing solely on physical aspects. Moreover, the higher prevalence in this study might also be related to the COVID-19 pandemic. During this period, many older adult individuals stayed indoors for extended periods, leading to reduced physical activity and functional decline, which likely contributed to increased frailty risk.

The study reveals that frailty in older adult individuals is influenced by multiple factors, with BMI, living arrangement, visit frequency, and smoking status being the primary contributors among community-dwelling older adult in this research. BMI is a key indicator of nutritional status and is widely used to assess the impact of various diseases in the older adult, such as hypertension, dyslipidemia, and chronic conditions. The findings show that being overweight or obese significantly increases frailty risk among the older adult. Obesity and excess weight, as metabolic conditions, can accelerate functional impairments and disabilities, aligning with observations in Pemecutan Village, Bali (26). Yingzhen Gu’s research (27) further highlights a positive correlation between frailty risk and BMI, noting that higher fat mass, reduced physical activity, and muscle loss are associated with greater frailty risk. Additionally, longer duration of obesity exacerbates this risk. Kai Guo’s study (28) also links elevated TyG-BMI (triglyceride-glucose body mass index) to rapid frailty progression, emphasizing the challenge for older adult individuals to maintain stable weight. Given the frequent need for medication to manage blood glucose and triglyceride levels among these participants, identifying an optimal TyG-BMI range and developing strategies to maintain it is crucial. Thus, BMI plays a significant role in frailty among community-dwelling older adult, underscoring the importance of community workers and caregivers providing targeted interventions and encouraging older adult individuals to monitor their BMI to prevent excessive weight gain and mitigate frailty risk.

Living arrangement and visit frequency are significant factors influencing frailty. Older adult individuals who live alone or receive few visits face a higher risk of frailty. Social isolation or loneliness can directly impact their physiology through neuroendocrine and immune system responses, often leading to conditions like depression and cardiovascular diseases. These individuals may also display personality traits such as low self-esteem and poor self-control, making them more susceptible to stress and increasing their risk of frailty and mortality. This aligns with findings from Hoogendijk et al. (11). A Singaporean study (29) further demonstrated that active social engagement can slow the progression of frailty, highlighting the importance of promoting social participation and addressing loneliness to prevent frailty. A large-scale UK survey (30) suggested a bidirectional relationship between frailty and loneliness: frailty may increase future loneliness, while lonely older adult individuals are more likely to be inactive, leading to reduced muscle mass and strength, and potentially impacting their eating habits and overall health. To support older adult individuals who live alone or are infrequently visited, options like senior apartments or community-based shared living spaces should be considered. Communities can organize regular visits or group activities by staff or volunteers, which not only improve physical functioning but also help build social networks and reduce loneliness.

Smoking significantly increases the risk of frailty in older adults, with smokers being 1.443 times more likely to develop frailty than non-smokers. Research by Lv et al. (31) indicates that lifelong smoking, influenced by genetic factors, is associated with frailty, suggesting that smoking is a causal factor. The harmful chemicals in cigarettes can damage multiple organs, contributing to both physical and mental health issues that increase the likelihood of frailty. Chronic inflammation and oxidative stress are key pathways linking smoking to frailty. This study aligns with the findings of Marcel et al. (32), showing that smoking impacts physical health as well as mental and social well-being. Smoking may alter neural pathways, increasing sensitivity to stress and contributing to conditions like depression or anxiety. Additionally, the social stigma surrounding smoking can lead to feelings of isolation and loneliness. Therefore, quitting smoking can improve physical health, enhance mental well-being, and increase social engagement in older adults. In addition to the previously mentioned factors, two key features identified in frailty risk prediction models warrant special attention: Type of Medication and Number of Chronic Diseases. Lv et al. (5) found that the cumulative effect of chronic diseases significantly raises the risk of frailty among the older adult. This study confirmed that the number of chronic diseases is a critical predictor of frailty. Moreover, an increased number of medications is strongly associated with frailty (OR = 1.156). Polypharmacy can lead to drug interactions, which may exacerbate frailty risk (33). The type of medication is also a critical factor, as a higher number of medications increases the likelihood of adverse reactions and interactions, potentially accelerating physical decline and raising the likelihood of frailty.

Machine learning, a widely used data mining tool, has been used to predict vulnerability risk in older adults. A study by Wang et al. (34) used a decision tree model to predict the risk of physical limitations in long-term care in older adults. Although these models are intuitive and easy to interpret, they tend to overfit and have lower predictive performance than other models. This is consistent with the results of this study, in which decision trees demonstrated their effectiveness in initial screening, but were less effective in predicting fatal risk. In contrast, the random forest model shows better generalization ability and is especially good at processing high-dimensional data. Wu’s study (35) confirmed the high accuracy and stability of random forest model in predicting decline trajectory, and affirmed its practicability in predicting vulnerability risk. The work of Han and Wang (36), and Noh (37) demonstrated the ability of XGBoost models to handle nonlinear problems and large-scale data, effectively capturing complex relationships between variables. In conclusion, both random forest and XGBoost models have their advantages in vulnerability risk prediction. These models will play an important role in future vulnerability risk assessment and health management for older people. By analyzing the main characteristics and influencing factors of vulnerability risk, it is helpful to deeply understand the degree of vulnerability risk, strengthen early prevention, and effectively mitigate its occurrence and development, so as to improve the quality of life of the older adult. The study also highlights the potential of machine learning technologies for use in healthcare. Provides new ideas for data analysis and forecasting in healthcare (36). These technologies can be widely used to predict and manage other health problems, thereby improving the efficiency and accuracy of the healthcare system. By promoting frailty risk prediction models based on machine learning, frailty in the older adult can be better addressed and healthy aging of the global older population can be promoted (2). The multi-dimensional intervention strategy emphasized in this study also provides an important reference for global public health policy making.

## 5 Limitation

This cross-sectional study does not establish causality between frailty and its associated risk factors, precluding the model from making prospective predictions about future frailty. Therefore, future research should incorporate longitudinal studies to elucidate the causal mechanisms and progression of frailty. Additionally, the study’s sample, drawn exclusively from eastern China, limits the generalizability of the findings to older adult populations in other regions. Moreover, the decision tree, random forest, and XGBoost models lack external validation. Future work should prioritize multi-center external validation to enhance model predictive performance and develop a frailty prediction model applicable to older adult individuals across the country.

## 6 Conclusion

In this research, BMI, living arrangements, visit frequency, and smoking status emerged as critical predictors of frailty among the older adult. A machine learning approach was utilized to forecast frailty risk within a community in eastern China. Ensemble models like random forest and XGBoost exhibited enhanced generalizability and robustness over single-model algorithms. Early identification of at-risk individuals and the implementation of personalized interventions can effectively slow frailty progression and improve quality of life in older age.

## Data Availability

The original contributions presented in the study are included in the article/supplementary material, further inquiries can be directed to the corresponding author.
